# What evidence syntheses reveal about PROSPERO, INPLASY, OSF, the Research Registry, and protocols.io: a meta-research study

**DOI:** 10.3389/frma.2026.1738112

**Published:** 2026-03-02

**Authors:** Raissa Tabosa Ferreira, Manuela Queiroz Reis, Maria Heloísa Pignataro Lange, Maycon Willian Fontes da Costa, Giovanna Kettlen Mendes Silva, Anne Beatriz Oliveira Lemos, Douglas Raphael Lela Dias, Maria de Lourdes Santos Viana, Ana Luiza Oliveira dos Santos, Ana Beatriz Maia Fernandes, Manuella Rocha Boaventura Pinheiro, Clarice Wanderley de Sousa, Kevin Henrique Azevedo Duarte, Gustavo Vicentis de Oliveira Fernandes, Carlos Marcelo da Silva Figueredo, Mario Vianna Vettore, Marcelo Marotta Araujo, Fabio Gamboa Ritto, João Vitor dos Santos Canellas

**Affiliations:** 1 Department of Dentistry, School of Health Sciences, University of Brasilia (UnB), Brasília, Brazil; 2 Missouri School of Dentistry & Oral Health, A. T. Still University, St. Louis, MO, United States; 3 School of Medicine and Dentistry, Griffith University, Gold Coast, QLD, Australia; 4 Department of Dentistry and Oral Health, Aarhus University, Aarhus, Denmark; 5 Department of Restorative Dentistry, Institute of Science and Technology, São Paulo State University, São José dos Campos, Brazil; 6 Department of Oral and Maxillofacial Surgery, College of Dentistry, University of Oklahoma, Oklahoma City, OK, United States; 7 Department of Research, INPLASY, Inc., Middletown, DE, United States

**Keywords:** evidence synthesis, INPLASY, Open Science Framework, PROSPERO, protocol, registration, scoping review, systematic review

## Abstract

**Background:**

This meta-research study examined how protocol registration information is reported in evidence syntheses published from 2020 to 2025 and registered in PROSPERO, INPLASY, OSF, the Research Registry, or protocols.io.

**Methods:**

We analyzed 4,750 studies covering various evidence synthesis types. Data were collected on registry use, reporting transparency, and accessibility features. Statistical comparisons included effect sizes with 95% confidence intervals.

**Results:**

PROSPERO remained the most widely used, with registrations from 70 countries. Among studies registered on non-PROSPERO platforms, more than 90% were found in INPLASY and OSF. Compared with PROSPERO, both registries demonstrated stronger reporting practices, with higher protocol status updates in INPLASY and more hyperlinks in OSF. However, a hyperlink did not always ensure public availability, as several OSF protocols required author authorization. Protocols in PROSPERO were associated with multiple publishers. In contrast, INPLASY protocols were more frequently linked to open-access journals, particularly those published by Frontiers and MDPI.

**Conclusion:**

Although PROSPERO remains the reference registry, INPLASY and OSF are playing an increasingly important role in promoting openness and accessibility. Researchers are encouraged to search multiple registries, especially PROSPERO, INPLASY, and OSF, before starting a new study to minimize the duplication of efforts.

**Systematic review registration:**

https://www.doi.org/10.37766/inplasy2025.6.0114, identifier INPLASY202560114.

## 1 Introduction

The first platform specifically created for the prospective registration of systematic review protocols was the International Prospective Register of Systematic Review (PROSPERO), maintained by the National Institute for Health Research (NIHR) in the United Kingdom (Booth et al., 2012). Since its launch in 2011, PROSPERO has become the leading international repository for systematic review protocols, particularly in the health sciences. However, prior to the COVID-19 pandemic, the rapid growth in the number of evidence syntheses led to unprecedented submission volumes and considerable delays in registration processing (Puljak, 2021).

Although PROSPERO remains the most widely used and recognized registry, it has some limitations. Funded by the United Kingdom (UK) government, submissions from the UK have historically been prioritized, with protocols from this region published earlier than those from other countries (Puljak, 2021). Furthermore, not all types of evidence syntheses are eligible for PROSPERO registration, and the registry does not prevent duplicate registrations on the same topic (Solla et al., 2021). Moreover, reliance on government funding raises questions about its long-term sustainability and operational capacity (Pieper and Rombey, 2022).

In response, alternative platforms have emerged, including INPLASY (Canellas et al., 2023), Open Science Framework (OSF) (Nosek et al., 2018), the Research Registry (Agha and Rosin, 2015), and protocols.io (Teytelman et al., 2016). With the emergence of these platforms, it has become possible to register additional types of studies and evidence syntheses that are not eligible for registration in PROSPERO.

Despite their growing adoption, little is known about the nature and scope of protocols registered in these platforms, including aspects such as thematic area, protocol status (e.g., ongoing, completed, or published), the method by which authors report their registration identifiers in resulting publications, and the journals in which the evidence syntheses are ultimately disseminated. This lack of information creates uncertainty regarding which repository is most appropriate for registering a given type of evidence synthesis and limits researchers' ability to identify ongoing or potentially overlapping studies.

Evidence syntheses are widely used by clinicians, policymakers, and the general public to inform healthcare and research decisions. To ensure their trustworthiness, methods must be transparent and reproducible, enabling readers to understand what was planned, what was conducted, and whether deviations occurred. Replicability is essential because undisclosed methodological changes, such as modifications to eligibility criteria, outcomes, or data synthesis strategies, can substantially affect conclusions and mislead decision-making. Accordingly, current best practices recommend the prospective development and public registration of protocols, together with clear documentation of amendments. Protocol registries support this process by providing time-stamped methodological records and facilitating the identification of ongoing or overlapping evidence syntheses. However, registries differ in scope, accessibility, and transparency features, and how these differences are reflected in published reporting remains uncertain. Clarifying these practices is therefore essential to strengthen reproducibility standards and reduce research waste.

This study provides new insights into the transparency and reporting practices of evidence syntheses published across scientific journals. By examining how information related to protocol registration is presented, this study contributes to a clearer understanding of the current level of adherence to best practices in evidence synthesis. The findings can inform editors, reviewers, and researchers about existing gaps in registration reporting, support improvements in editorial and methodological standards, and ultimately promote greater transparency and reproducibility in scientific research.

Understanding how registration practices are reported in published studies is essential. Therefore, this meta-research aimed to evaluate how protocol registration information is presented in evidence syntheses registered in PROSPERO, INPLASY, OSF, the Research Registry, and protocols.io. In addition, we compared evidence syntheses registered in PROSPERO with the other registries, considering factors such as journal impact factor, thematic area, and citation patterns, and using a large sample of articles published between 2020 and 2025.

## 2 Materials and methods

This was a methodological meta-research study designed prospectively and registered in the International Platform of Registered Systematic Review and Meta-Analysis Protocols (INPLASY) under the registration code INPLASY202560114.

We included articles that (1) are classified as a systematic review, scoping review, mapping review, umbrella review, meta-research, integrative review, and narrative review; (2) mention protocol registration in PROSPERO, INPLASY, OSF, the Research Registry, or protocols.io; and (3) are published between June 2020 and June 2025. No language restrictions were applied to the inclusion of evidence syntheses. Conference abstracts, preprints, expert opinions, and Cochrane reviews were excluded.

We conducted a systematic search using different electronic databases and other sources. MEDLINE via PubMed, Embase, and Web of Science databases were searched. In addition, a manual page-by-page examination was performed in the following websites: journals.sagepub.com, frontiersin.org, onlinelibrary.wiley.com, lww.com, nature.com, sciencedirect.com, tandfonline.com, www.mdpi.com, and inplasy.com. The detailed search strategy used for screening published evidence syntheses registered in the five major registries is available in the protocol available on http://doi.org/10.37766/inplasy2025.6.0114.

Two reviewers independently screened the retrieved articles at the title and abstract stage and subsequently at full-text review. Any discrepancies were resolved through discussion with a third reviewer. Information was extracted from the published articles and their corresponding protocols. Each included publication corresponded to a single registry protocol. When multiple publications referenced the same protocol ID, only the primary article was included to maintain a one-to-one correspondence between studies and protocols. All records were exported into EndNote 2025, where duplicates were first removed automatically by the software and then verified manually to ensure complete deduplication. We prespecified that if more than 1,800 records were retrieved from a single registry, simple random sampling would be applied at the title/abstract stage. This situation occurred only for PROSPERO. We calculated the sample size required for a representative random sample from 31,960, assuming a 95% confidence level, a conservative proportion of 0.5, and a 2.5% margin of error. After applying the finite population correction, we obtained an adjusted estimate of approximately 1,470 records. The final sample of 1,495 records, therefore, exceeded the minimum requirement for representativeness. All PROSPERO-linked records were numbered, and a random number table was used to select records for screening until the target sample size (approximately 1,800 records) was reached. For INPLASY, OSF, the Research Registry, and protocols.io, the total number of records did not exceed this threshold.

Data extracted from the included studies included the following information: title of the article; type of evidence synthesis (e.g., systematic review, scoping review, umbrella review, etc.); journal of publication (including the most recent Journal Impact Factor and the publisher name); journal categories; date of final publication as reported in the article or automatically indexed by EndNote; country of the corresponding author; name of the registry where the protocol was registered; registration ID; source of funding (Yes/No); existence of a peer-reviewed version of the protocol (Yes/No); if yes, the name of the journal; and hyperlink to the registration report (Yes/No, protocol url or DOI). The journal impact factor was extracted from Journal Citation Reports™ 2025 (Clarivate Analytics). A preliminary calibration phase was implemented before full data extraction to ensure consistent extraction results among reviewers. Inter-rater reliability for the primary categorical variables, including type of evidence synthesis, funding status, hyperlink availability, and protocol status, was measured using pairwise Cohen's kappa. For continuous variables, such as the journal impact factor and the registration-to-publication interval, intraclass correlation coefficients (ICCs) were calculated.

In addition, we accessed the protocols referred to in the manuscripts to extract the following information: current protocol status (i.e., whether the status was updated) and registration date (used to calculate the mean time from registration to publication).

We categorized all data and tabulated them descriptively to pinpoint similarities and differences between the evidence syntheses registered in different platforms.

The selection process is illustrated using the PRISMA flow diagram, while the characteristics of the included overviews are summarized descriptively and presented narratively through tables and figures. The data were descriptively analyzed. We quantitatively compared continuous and dichotomous variables from evidence syntheses registered in PROSPERO and in each of the other platforms by calculating risk ratios (RRs) and standardized mean differences (SMDs), each with corresponding 95% confidence intervals. All analyses were conducted as pairwise comparisons, with RRs and SMDs computed separately for each registry vs. PROSPERO. No pooled “non-PROSPERO” comparator group was created, and no aggregate effect size combining multiple registries was used. The nominal variables representing the nominal categories of the included studies were processed to generate a treemap. All statistical analyses were performed using the R software version 4.5.1 (2025-06-13) for the Mac OS X computer system.

In response to peer-review comments, we conducted *post-hoc* exploratory analyses to assess whether registry-related differences were attributable to underlying study characteristics. We conducted two *post-hoc* multivariable linear regression analyses. The first model examined time from protocol registration to publication as the dependent variable; the second model evaluated the journal impact factor (JIF). In both models, registry served as the primary exposure, and review type, country of origin, and funding status were included as covariates. Adjusted marginal means and pairwise contrasts vs. PROSPERO were obtained using the *emmeans* package, with Dunnett correction for multiple comparisons. These analyses aimed to quantify the independent contribution of registry choice beyond major study-level characteristics.

## 3 Results

**Figure 1:**
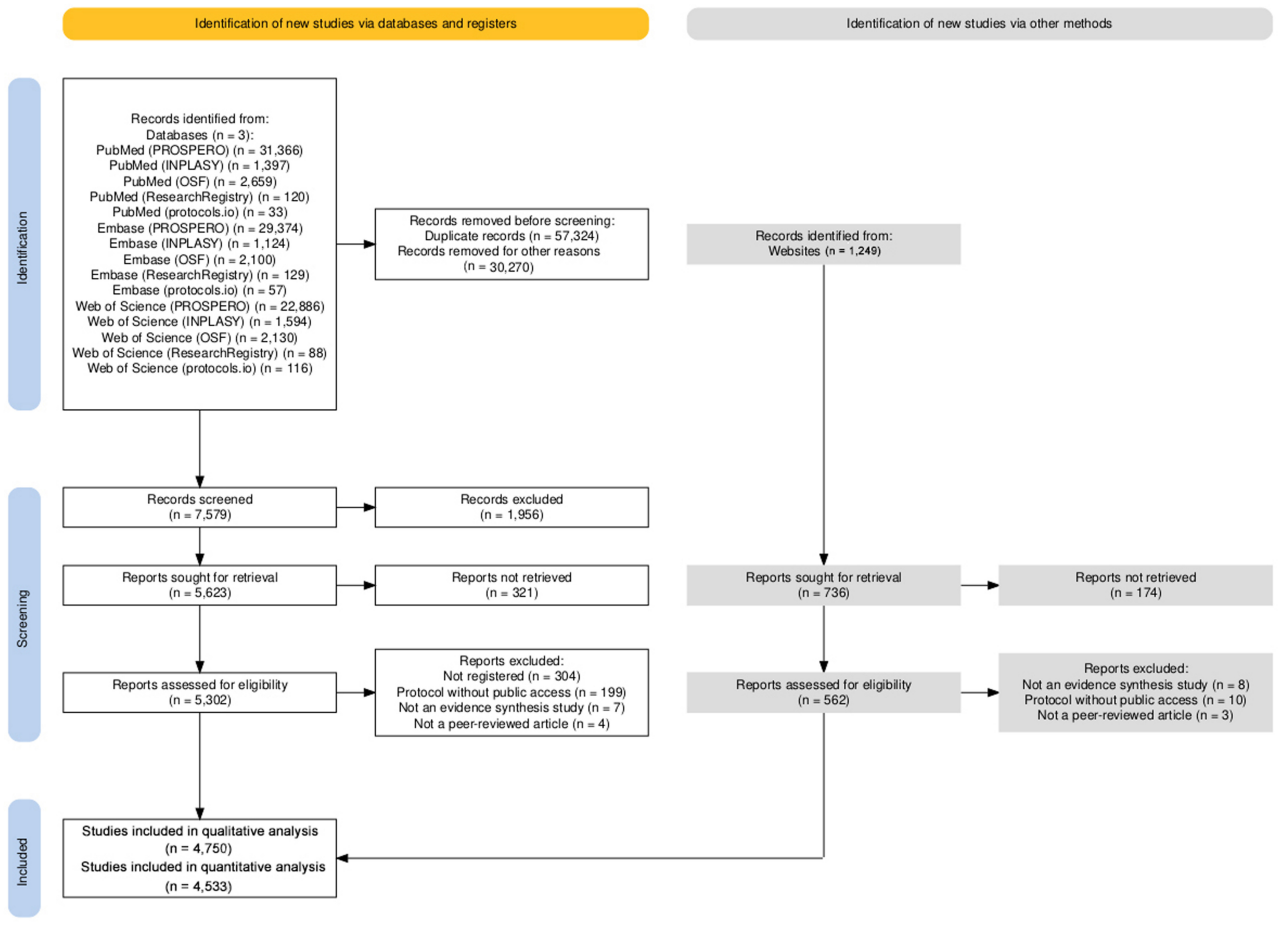
Flowchart illustrating the selection process of eligible studies. The final dataset comprises 4,750 unique evidence syntheses linked to 4,750 distinct protocols.

The electronic search identified 37,849 reports, of which 31,960 were from PROSPERO, 2,960 from OSF, 2,370 from INPLASY, 349 from the Research Registry, and 210 from protocols.io at the title and abstract screening stage. Owing to the large number of studies registered in PROSPERO, a random sample of articles was generated, resulting in the exclusion of 30,270 reports. Of the 7,579 reports reviewed in full text, 4,788 met the eligibility criteria (Figure 1). Consequently, this meta-research included 4,750 articles in the quantitative analysis and 4,533 in the qualitative analysis (See Supplementary Tables 1, 2).

A total of nine types of evidence synthesis were reviewed and included in this meta-research. The most frequent type was the systematic review with meta-analysis, accounting for 48% of all evidence syntheses. Table 1 presents the list of different types of evidence syntheses by registry.

**Table 1:** Number of published and registered articles across platforms by type of evidence synthesis study (1 June 2020–1 June 2025).

**Type of evidence synthesis study**	**PROSPERO *n*(f)**	**INPLASY *n*(f)**	**OSF *n*(f)**	**The Research Registry *n*(f)**	**protocols.io *n*(f)**
Systematic review	501 (33.51%)	274 (16.50%)	334 (26.59%)	92 (31.19%)	9 (20.45%)
Systematic Review + Meta-analysis	921 (61.60%)	1,171 (70.54%)	405 (32.24%)	168 (56.95%)	23 (52.27%)
Systematic review + network meta-analysis	41 (2.74%)	125 (7.53%)	4 (0.31%)	11 (3.73%)	1 (2.27)
Scoping review	8 (0.53%)	63 (3.79%)	492 (39.17%)	14 (4.75)	10 (22.73%)
Umbrella review	21 (1.40%)	20 (1.2%)	13 (1.03%)	2 (0.68%)	0 (0%)
Mapping review	0 (0%)	4 (0.24%)	3 (0.23%)	0 (0%)	0 (0%)
Integrative review	1 (0.06)	1 (0.06%)	2 (0.15%)	0 (0%)	0 (0%)
Metaresearch study	0 (0%)	1 (0.06%)	2 (0.15%)	0 (0%)	1 (2.27%)
Narrative review	2 (0.13)	1 (0.06%)	1 (0.07%)	8 (2.71)	0 (0%)
Total	1,495 (100%)	1,660 (100%)	1,256 (100%)	295 (100%)	44 (100%)

n, number of studies; f, frequency.

**Figure 2:**
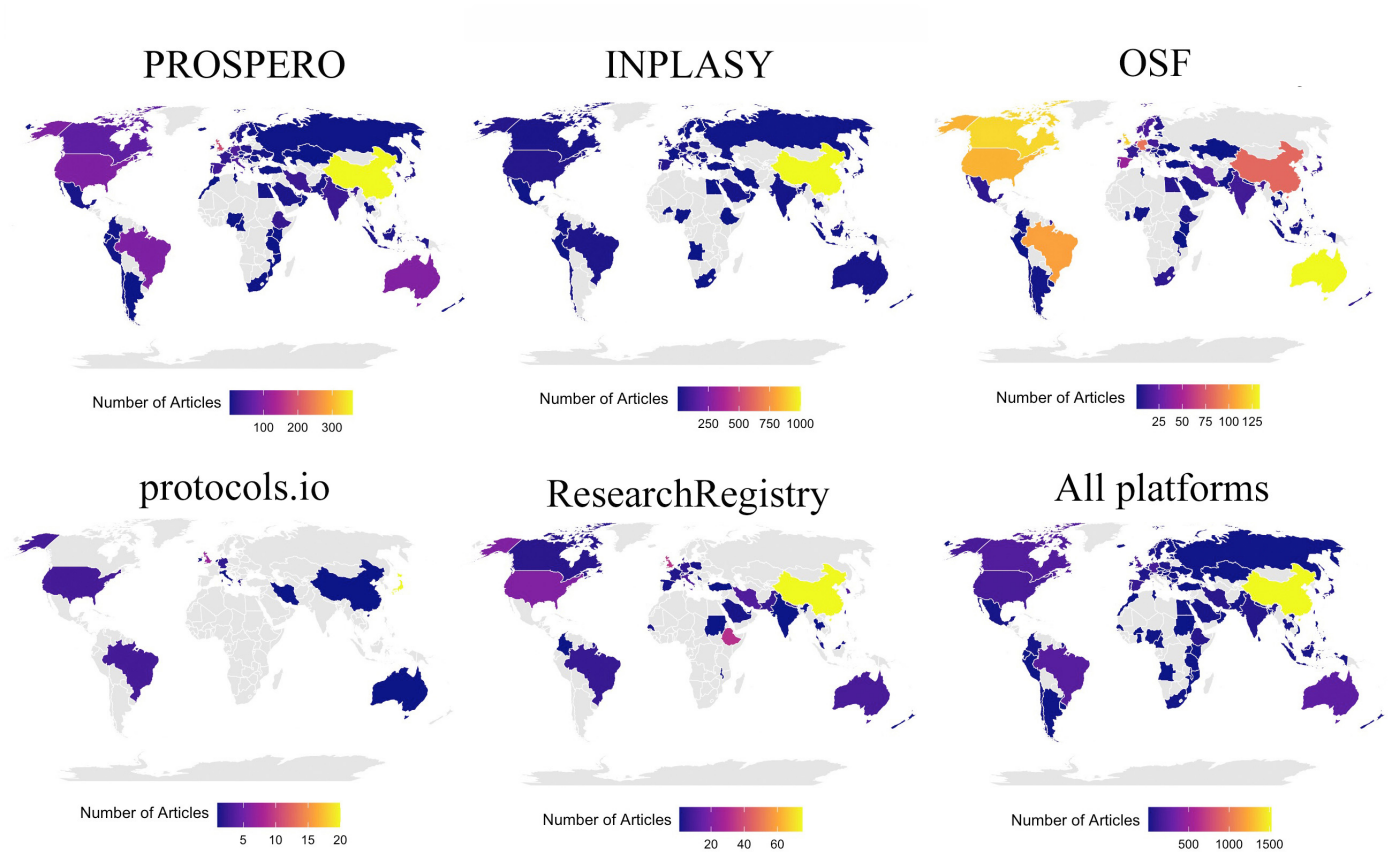
Choropleth map of the included article frequency by country.

The evidence syntheses registered in PROSPERO originated from 70 different countries, most of which were from China (25%), followed by the United Kingdom (13%), Australia, Brazil, and the United States (6% each). Evidence syntheses registered in INPLASY came from 58 countries, with the majority from China (64%), while the remaining articles were evenly distributed across continents. Evidence syntheses registered in OSF originated from 68 countries, primarily from Australia, Canada, the United Kingdom, the United States, and Brazil (approximately 9% each). Submissions to the Research Registry originated from 37 countries, most frequently from China (26%), followed by the United Kingdom and Ethiopia (11%). Finally, evidence syntheses registered in protocols.io originated from 12 countries, predominantly from Japan (46%), followed by the United Kingdom (16%). When considering all registries, China emerged as the country with the most evidence syntheses published in the last 5 years (33%), followed by the United Kingdom (8%), the United States, and Australia (5% each). A choropleth map illustrating the frequency of evidence syntheses by country is presented in Figure 2.

**Figure 3:**
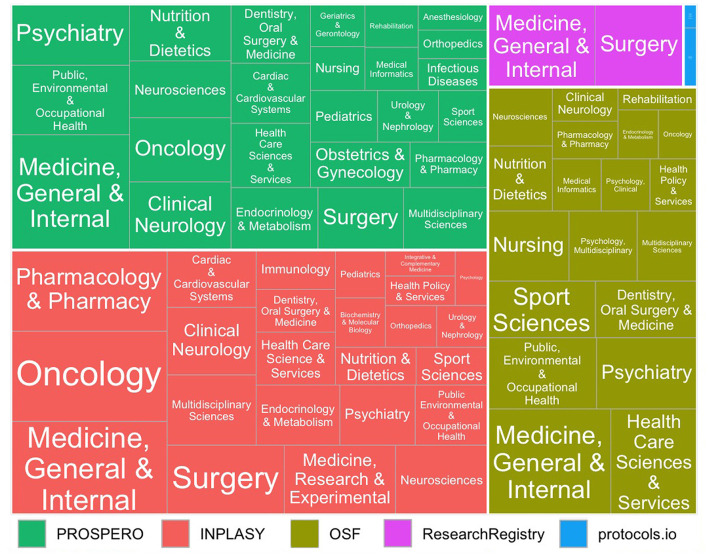
Treemap of journal categories identified among 225 included categories. Larger cells represent categories with higher frequency across the platforms PROSPERO, INPLASY, OSF, the Research Registry, and protocols.io.

The evidence syntheses were published in journals classified in 193 different categories. Medicine, General & Internal was the most frequently represented category across all databases. Figure 3 presents a treemap illustrating the most frequent thematic areas identified in each registry.

**Figure 4:**
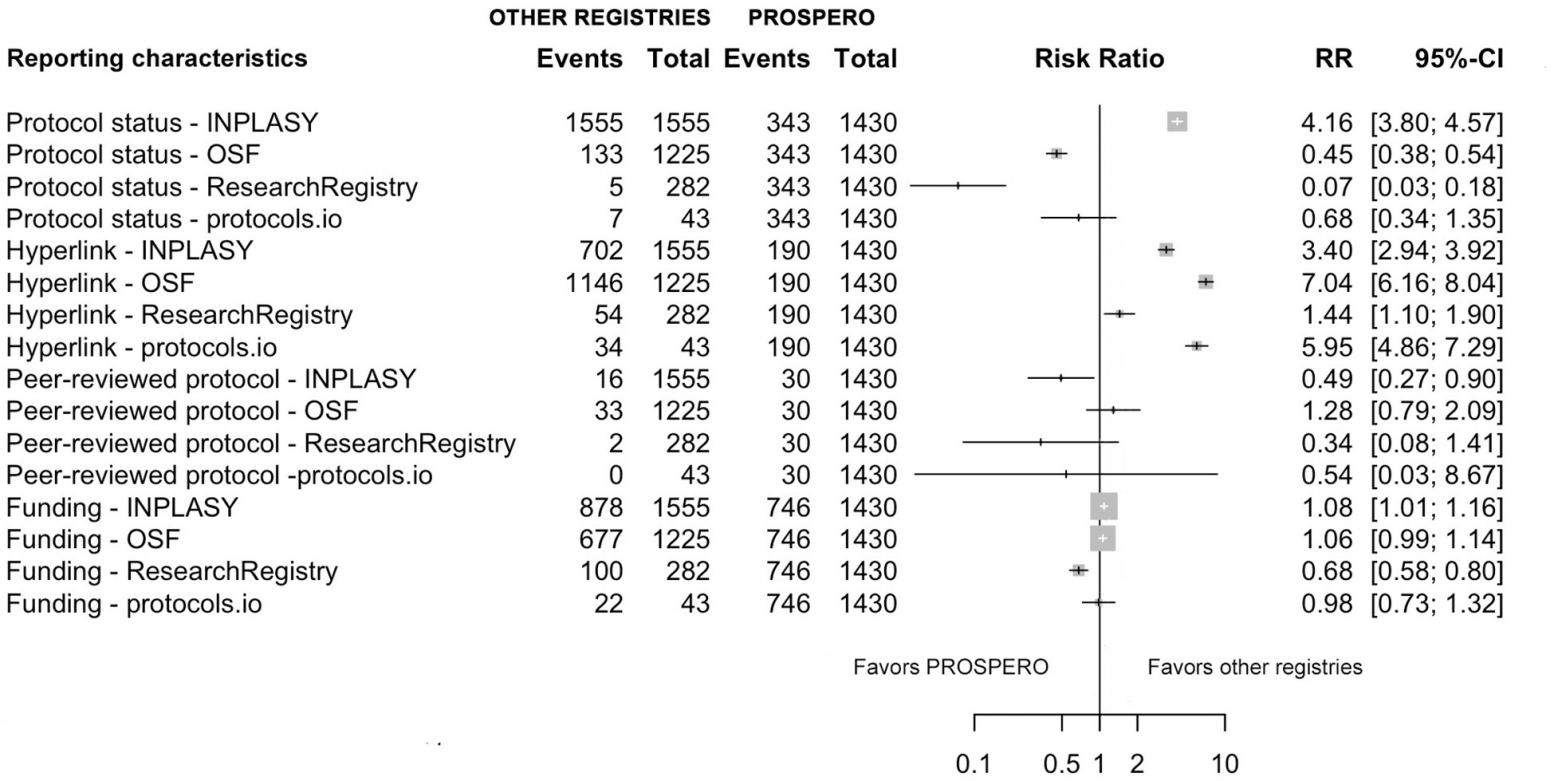
Comparison of reporting characteristics between PROSPERO and other registries. Risk ratio with 95% confidence intervals for key reporting features, including protocol status, hyperlink availability, peer review, and funding disclosure. Each line in the forest plot represents an independent pairwise comparison between PROSPERO and non-PROSPERO registries. No combined analysis of non-PROSPERO registries was performed.

**Figure 5:**
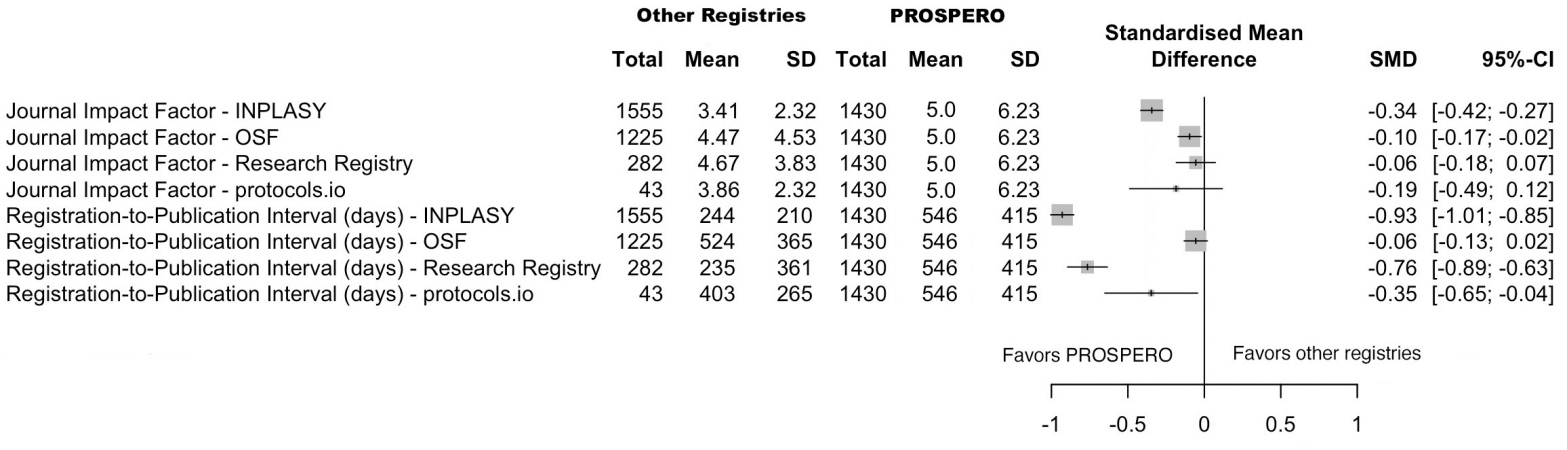
Standardized mean differences with 95% confidence intervals comparing journal impact factors and registration-to-publication intervals between PROSPERO and other registries.

The dichotomous and continuous variables of reporting characteristics of evidence syntheses registered in PROSPERO were compared with those in other registries (INPLASY, OSF, the Research Registry, and protocols.io) (Figures 4, 5).

For dichotomous variables, the pooled RR and their 95% confidence intervals indicated marked variation across characteristics. Protocol status was significantly more frequently reported in INPLASY (RR = 4.16, 95% CI 3.80–4.57) compared with PROSPERO. Hyperlink availability was higher in other registries, INPLASY (RR = 3.40, 95% CI 2.94–3.92), OSF (RR = 7.04, 95% CI 6.16–8.04), the Research Registry (RR = 1.44, 95% CI 1.10–1.90), and protocols.io (RR = 5.95, 95% CI 4.86–7.29), suggesting better accessibility of protocols registered in non-PROSPERO registries. Conversely, the publication of peer-reviewed protocols was less frequent across other platforms, but only INPLASY showed a statistically significant reduction, RR = 0.49 (95% CI 0.27–0.90). Regarding funding information, a small but significant difference is observed in INPLASY (RR = 1.08, 95% CI 1.01–1.16), while other non-PROSPERO registries showed similar reporting rates. Overall, six reporting characteristics were statistically significantly better in non-PROSPERO registries, and four reporting characteristics were statistically significantly better in PROSPERO. The remaining dichotomous reporting characteristics showed no statistically significant differences between PROSPERO vs. non-PROSPERO registries.

For continuous variables, the pooled SMD with 95% CI showed that PROSPERO registrations are linked to higher journal impact factors. However, they are also associated with longer publication timelines.

**Figure 6:**
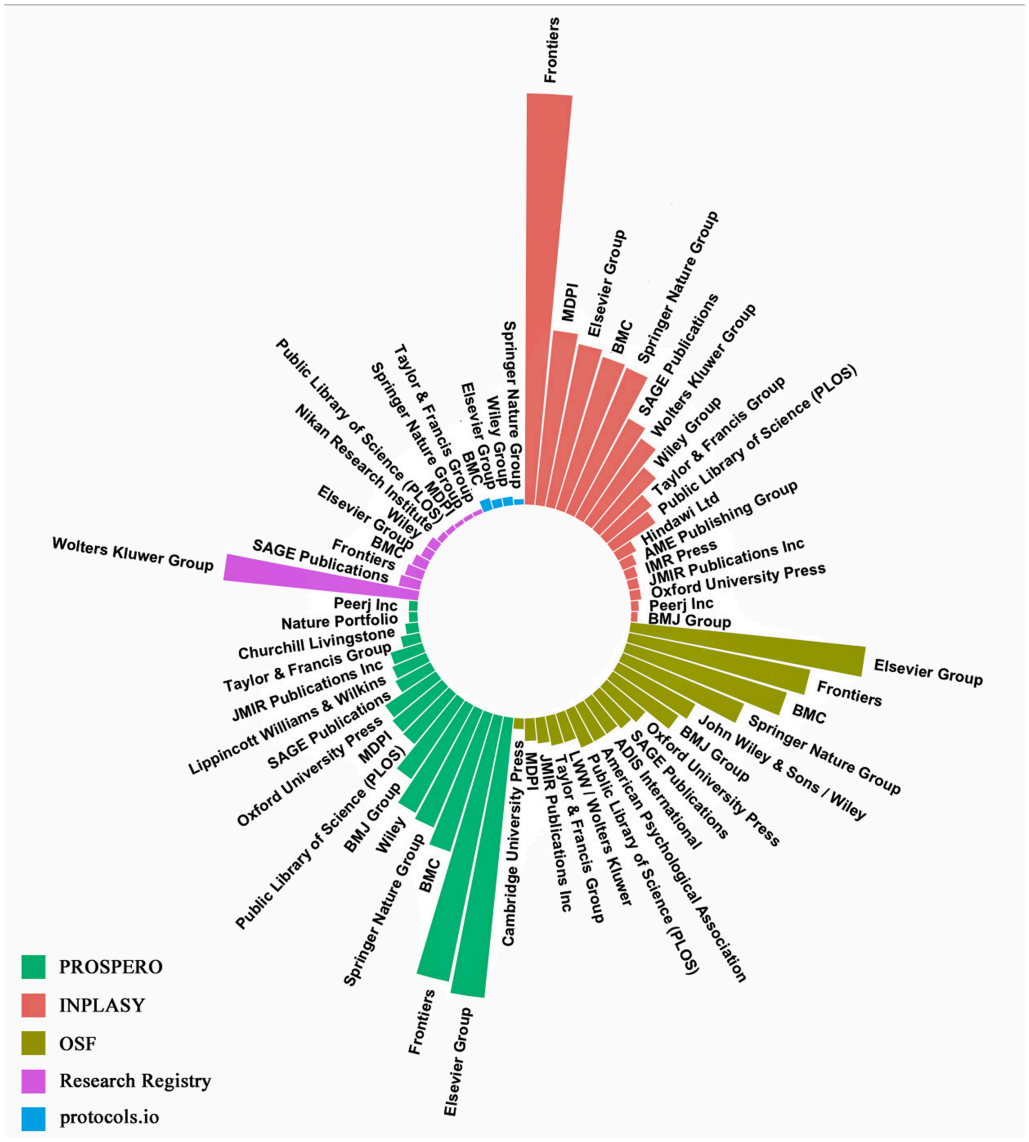
Distribution of evidence synthesis publications by publisher and registry platform. Each bar represents the number of evidence syntheses published by a specific publisher, grouped by the registry in which their protocols were registered.

Finally, Figure 6 illustrates the distribution of evidence synthesis publications across different publishers by the platform where the study was registered. A distinct publishing pattern among different registries is noted. INPLASY had the highest concentration of publications in Frontiers, accounting for the most significant volume among all registry–publisher combinations. PROSPERO-registered studies were published across a more diverse set of publishers, including Cambridge University Press, Springer Nature Group, Elsevier Group, and BMJ Group. Evidence syntheses registered in OSF were frequently published by Elsevier, Frontiers, BMC, and Wiley, whereas those from the Research Registry showed a marked presence in SAGE Publications and Wolters Kluwer Group. Publications associated with protocols.io were minimal and spread among a few publishers.

Finally, inter-rater reliability analysis indicated substantial to almost perfect agreement for the categorical variables assessed. Kappa coefficients ranged from κ = 0.653 to κ = 0.840, and observed agreement between reviewers ranged from 0.84 to 0.96. Continuous variables demonstrated strong reproducibility; journal impact factor showed excellent reliability (ICC = 0.999), and the registration-to-publication interval demonstrated good reliability (ICC = 0.733). These findings indicate that data extraction was consistent across reviewers and reinforce the robustness of the final dataset.

### 3.1 *Post-hoc* exploratory analyses

#### 3.1.1 Adjusted publication timelines

After adjusting for review type, country, and funding, registry-related differences in time to publication persisted. Compared with PROSPERO (adjusted mean = 513 days), INPLASY (286 days; Δ = −226.8, *p* < 0.0001), OSF (437 days; Δ = −76.2, *p* = 0.0001), and the Research Registry (262 days; Δ = −251.1, *p* < 0.0001) showed significantly shorter intervals. The difference for protocols.io (400 days; Δ = −112.5) was not statistically significant (*p* = 0.1601).

#### 3.1.2 Adjusted journal impact factor

After similar adjustment, differences in JIF across registries were minimal. Relative to PROSPERO (adjusted mean = 4.33), INPLASY showed a slightly lower adjusted JIF (Δ = −0.91, *p* < 0.0001), while OSF (Δ = 0.22, *p* = 0.73) and protocols.io (Δ = −0.11, *p* = 1.00) did not differ significantly. The Research Registry showed a modest increase (Δ = 0.84, *p* = 0.03). Overall, no substantial registry-driven variation in JIF was detected.

## 4 Discussion

The present meta-research provides a comprehensive evaluation of protocol registration practices across PROSPERO, INPLASY, OSF, the Research Registry, and protocols.io, based on evidence syntheses published in different journals and registered with these platforms. The findings reveal insights into how these platforms register evidence syntheses, their geographic reach, the types of evidence syntheses they host, and, especially, how the registration information is reported in the published articles. Although it is desirable, none of the existing protocol registries provide methodological oversight or peer review of submitted records.

From this perspective, Cochrane reviews were excluded because they are subject to mandatory peer review and centralized editorial oversight, in contrast to protocols registered in PROSPERO or other evaluated registries, which do not include peer review in their registration processes. Consequently, Cochrane reporting practices reflect organizational standards rather than the authors' engagement with registry features. Including Cochrane reviews would artificially increase PROSPERO's reporting performance and introduce bias into cross-registry comparisons. Their exclusion allows for more valid and comparable inferences across platforms.

PROSPERO, launched in 2011 (Booth et al., 2011), remains the most widely used platform for evidence synthesis registrations, particularly systematic reviews, as demonstrated by the large number of studies screened in the electronic databases. Its leadership status is reflected in its greater authorship participation worldwide, with protocols originating from 70 countries and significant contributions from China, the United Kingdom, Australia, and the United States. Unfortunately, PROSPERO does not accept all types of evidence syntheses, excluding, in particular, scoping reviews. Developing a scoping review protocol enhances the quality, reproducibility, and trustworthiness of the review.

In this context, platforms such as INPLASY and OSF have emerged as alternatives to scoping review registration. Our study identified a large number of scoping reviews registered in non-PROSPERO databases, especially in OSF. Additionally, as studies without health-related outcomes are not eligible for registration in PROSPERO, we identified numerous evidence syntheses measuring sports performance and other non-health-related outcomes that were registered instead in OSF and INPLASY, highlighting the contribution of non-PROSPERO registries to various thematic areas.

Our study identified a longer publication interval between registration and publication for PROSPERO-registered evidence syntheses, which could be explained by several factors. PROSPERO's status as the oldest and most recognized registry may encourage authors to identify it as a higher-quality platform and, consequently, more appropriate for complex and elaborated studies. However, the methodological quality of the protocol may not be influenced by the registry, since none of the available platforms have conducted a peer review of submitted protocols. A total of 81 studies reported a peer-reviewed protocol, corresponding to only 1.7% of our sample. This finding indicated that, although it is recommended to publish protocols in peer-reviewed journals to increase elaboration and report quality (Allers et al., 2018), this practice is not widely used by the scientific community.

Puljak (2021) reported delays in PROSPERO registration, especially before and during the COVID-19 pandemic. The author has suggested that a longer timeline between registration and publication can reduce transparency. Our analysis revealed that studies registered in PROSPERO took longer from registration to publication. Although the fast processing times of emerging platforms can affect timelines between registration and publication, other factors, such as differences in the editorial practices of scientific journals, should be more relevant. For instance, most protocols registered in PROSPERO tend to be published in Elsevier journals, which generally have longer editorial timelines than those of the Frontiers group (Csomós and Farkas, 2023), where a higher concentration of INPLASY protocols is observed.

Finally, an important structural factor influencing these findings is that PROSPERO requires prospective registration, whereas other platforms may allow retrospective submissions. This can shorten the registration-to-publication interval in non-PROSPERO registries, as some protocols may be registered only after substantial progress, or even completion, of the evidence synthesis. Because we could not determine retrospectiveness for individual records, the magnitude of this effect remains uncertain, and differences in timelines should therefore be interpreted with caution.

Hu et al. (2021) identified significant inconsistencies between the eligibility criteria reported in protocols and those described in the final published systematic reviews. Based on these findings, it could be suggested that access to registered protocols be improved and facilitated to ensure greater transparency. Our findings identify a significant variation in how the protocol registration details are reported across platforms. Non-PROSPERO registries, particularly INPLASY and OSF, showed higher rates of hyperlinks and DOI availability in published articles, which can potentially improve research transparency. However, these indicators must be interpreted with caution. Several OSF-registered protocols were not publicly accessible, as they required author authorization to view the files. This limitation demonstrates that the presence of a hyperlink does not guarantee meaningful transparency if the underlying protocol cannot be accessed. In contrast, INPLASY's higher hyperlink rates correspond to consistently open and publicly available records. Thus, while hyperlink availability is a useful marker of reporting quality, actual accessibility ultimately depends on the platform's visibility settings and authors' sharing practices.

The evidence syntheses' geographical distribution confirms the global status of the analyzed platforms, with China emerging as the leading contributor. China's predominance in INPLASY registrations may be understood in light of several contextual factors. First, the country has become one of the leading global contributors to evidence synthesis. Many Chinese universities and funding agencies strongly encourage the registration of evidence synthesis protocols, which aligns with the high volume of submissions observed in both PROSPERO and INPLASY. Second, INPLASY offered a long-standing reduced-fee policy for authors affiliated with Chinese institutions, a feature that likely further incentivized uptake among researchers from that country. Finally, during its first 3 years of operation, INPLASY implemented a set of targeted marketing actions aimed specifically at Chinese researchers and academic institutions. Together, these factors help explain the substantial proportion of protocol registrations originating from China in the period analyzed. In this context, it is important to highlight the need to support multiple registries to accommodate diverse research groups worldwide.

The diversity of journal categories and publishers in which registered evidence syntheses are published further reflects the multidisciplinary and international nature of the registries. Evidence syntheses registered in PROSPERO appear more broadly dispersed across publishers. In contrast, evidence syntheses registered in INPLASY are concentrated in journals such as Frontiers and MDPI, suggesting a possible focus on open-access journals. Additionally, it has been observed that INPLASY is present in leading medical and scientific journals, reflecting their recent growing recognition as a valid platform for evidence synthesis registration (Greenhalgh and McKee, 2025; Wang et al., 2025). Moreover, registrations in non-PROSPERO platforms have increased, particularly in INPLASY and OSF, indicating broader adoption of these alternative databases.

The study also highlights ongoing challenges related to duplication and overlap in systematic reviews. PROSPERO does not currently prevent duplicate registrations on the same topic, and this limitation likely applies to the other platforms as well. Beresford et al. (2022) compared PROSPERO records using the Population, Intervention/Exposure, Comparator, Outcome, Study Design (PICOS) elements. They found that registration of similar and duplicate COVID-19 systematic reviews occurred frequently. Given the large number of evidence syntheses registered in non-PROSPERO platforms, initial checks for similar studies in other registries are strongly recommended to reduce unintended duplication of research.

Contrarily, Banno et al. (2022) found a very low proportion of systematic review protocols registered outside PROSPERO, concluding that it might not be necessary to search non-PROSPERO protocols to prevent duplicate effort. However, the authors analyzed included protocols registered between 2011 and 2020. Our meta-research found a substantial number of systematic reviews registered on non-PROSPERO platforms, particularly between 2020 and 2025, following the launch of INPLASY in 2020 and the growing popularity of OSF in recent years. Therefore, it is recommended to search not only the PROSPERO database but also non-PROSPERO databases, such as INPLASY and OSF, before starting a new evidence synthesis. Additionally, our results highlight the critical need for improved coordination among registries and for enhanced mechanisms to identify overlapping studies, thereby minimizing research waste.

Furthermore, PROSPERO's reliance on government funding raises concerns about sustainability and long-term operational capacity, which may have catalyzed the emergence and growth of alternative platforms. As a result of these concerns, it is possible to find evidence syntheses that describe two different registries, PROSPERO and one for-profit organization, such as INPLASY (Park et al., 2025).

A key strength of this meta-research study is its large and diverse sample, including data extracted from 4,750 articles, and incorporating a wide range of evidence synthesis types, such as systematic and scoping reviews. This inclusiveness reflects the evolving landscape of evidence synthesis methodologies and the expanding scope of protocol registration. The comprehensive search across multiple databases and manual screening of prominent journal platforms enhance the robustness of the findings, including articles not identified in the main databases.

The *post-hoc* analyses indicate that shorter publication timelines observed for INPLASY, OSF, and the Research Registry persist even after accounting for major study-level characteristics, suggesting that registry-specific operational factors may influence the speed from registration to publication. The non-significant difference for protocols.io likely reflects a limited sample size. In contrast, adjusted analyses showed only small differences in journal impact factor across registries, suggesting that JIF is largely determined by the characteristics of the included studies rather than the registry used. While residual confounding cannot be fully excluded, the combined findings support the interpretation that registry choice has a modest influence on journal impact factor but a more pronounced independent association with publication timelines.

Nevertheless, this study has some limitations. Our analysis focused on reporting characteristics and publication metrics, but did not assess the methodological quality of the protocols or the evidence syntheses derived from them, which would be valuable for future research. Additionally, the functionality of the platforms and the total number of protocols registered in each of them were not analyzed. Due to the large number of evidence syntheses published in PROSPERO, our research team was unable to analyze all of them; therefore, a representative random sample of the screened articles was selected for comparison with studies registered in other databases.

Furthermore, although one lead author had a declared conflict of interest related to INPLASY, several safeguards were implemented to minimize potential bias. All screening, eligibility assessment, and data extraction procedures were carried out independently by researchers with no affiliation to INPLASY, using duplicate extraction and adjudication to ensure reliability. The conflicted author did not participate in data extraction or study selection, and all analyses adhered to a predefined protocol, reducing the influence of subjective judgment on the results.

Overall, we observed marked differences among the main registries for evidence synthesis protocols. PROSPERO, in particular, has remained the reference platform in this field, with many of its registered studies appearing in journals published by Springer Nature, Elsevier, and the BMJ Group. By contrast, other registries, particularly OSF and INPLASY, perform better in terms of transparency, as they more frequently include DOIs, direct links, and updated protocol statuses. Given the small sample size, the Research Registry and protocols.io appear to make a smaller contribution in this field. Taken together, these findings reinforce the need for better coordination among registries to reduce duplication of projects. In practical terms, we suggest that researchers search PROSPERO, INPLASY, and OSF before initiating a new evidence synthesis to avoid overlap and duplication of effort.

## Data Availability

The original contributions presented in the study are included in the article/Supplementary material, further inquiries can be directed to the corresponding author.
